# Signaling – transcription interactions in mouse retinal ganglion cells early axon pathfinding –a literature review

**DOI:** 10.3389/fopht.2023.1180142

**Published:** 2023-05-17

**Authors:** Raluca Paşcalău, Tudor Constantin Badea

**Affiliations:** ^1^ Research and Development Institute, Transilvania University of Braşov, Braşov, Romania; ^2^ Ophthalmology Clinic, Cluj County Emergency Hospital, Cluj-Napoca, Romania; ^3^ National Center for Brain Research, Institutul de Cercetări pentru Inteligență Artificială, Romanian Academy, Bucharest, Romania

**Keywords:** optic nerve, retinal ganglion cells, transcription, development, optic stalk

## Abstract

Sending an axon out of the eye and into the target brain nuclei is the defining feature of retinal ganglion cells (RGCs). The literature on RGC axon pathfinding is vast, but it focuses mostly on decision making events such as midline crossing at the optic chiasm or retinotopic mapping at the target nuclei. In comparison, the exit of RGC axons out of the eye is much less explored. The first checkpoint on the RGC axons’ path is the optic cup - optic stalk junction (OC-OS). OC-OS development and the exit of the RGC pioneer axons out of the eye are coordinated spatially and temporally. By the time the optic nerve head domain is specified, the optic fissure margins are in contact and the fusion process is ongoing, the first RGCs are born in its proximity and send pioneer axons in the optic stalk. RGC differentiation continues in centrifugal waves. Later born RGC axons fasciculate with the more mature axons. Growth cones at the end of the axons respond to guidance cues to adopt a centripetal direction, maintain nerve fiber layer restriction and to leave the optic cup. Although there is extensive information on OC-OS development, we still have important unanswered questions regarding its contribution to the exit of the RGC axons out of the eye. We are still to distinguish the morphogens of the OC-OS from the axon guidance molecules which are expressed in the same place at the same time. The early RGC transcription programs responsible for axon emergence and pathfinding are also unknown. This review summarizes the molecular mechanisms for early RGC axon guidance by contextualizing mouse knock-out studies on OC-OS development with the recent transcriptomic studies on developing RGCs in an attempt to contribute to the understanding of human optic nerve developmental anomalies. The published data summarized here suggests that the developing optic nerve head provides a physical channel (the closing optic fissure) as well as molecular guidance cues for the pioneer RGC axons to exit the eye.

## 1 Introduction

The mammalian retina comprises five classes of neuronal cells (1): the photoreceptors transduce light into an electrical signal and transmit it in the outer plexiform layer to bipolar cells. At this level lateral interactions are provided by horizontal cells. In the inner plexiform layer (IPL), the bipolar cells connect to the retinal ganglion cells (RGCs), which send the visual information in the form of nerve spikes to the retinorecipient nuclei in the brain. At IPL level, amacrine cells assist in retinal computation by a variety of inhibitory and excitatory lateral connections with bipolar cells and RGCs. Until now, more than 40 RGC types have been identified, which receive various combinations of signals from the approximately 70 types of interneurons so that they extract distinct visualqualities (2–10). RGCs of the same type form anatomical and functional mosaics within the retina, namely the dendritic arbors of a given RGC type tile the retina uniformly and their receptive fields sample the visual scene and extract specific visual features (11–13). As a result, every point in the visual field is reported to the brain through multiple parallel channels (14) dedicated to different visual modalities such as contrast, color, or motion (1, 2, 15–18) and the brain receives a number of parallel images of the world (3). The anatomical basis of this connection is the optic nerve, a fascicle of RGC axons linking the retina to the brain. In the last decades, the field of developmental neuroscience has predominantly focused on the study of cell type specification, especially encouraged by the advent of single-cell RNA sequencing tools (19–21). Sending an axon towards the optic disk and through the optic nerve is a defining feature of all RGCs, regardless of the cell type. It is one of the earliest developmental events, occurring right after RGCs differentiate (22–25), at a time when RGC types are not yet specified (26). Although numerous papers have reviewed RGC axon guidance mechanisms (27–36) – most of them are focused on population - level axon steering events such as chiasm crossing and retinotopic mapping at the targets while the determinants of RGC axon emergence and optic nerve formation are far less explored.

The need to more comprehensively approach this subject is enforced by the increase in frequency of optic nerve development anomalies in humans. A significant cause of congenital blindness, human optic nerve developmental anomalies are a heterogeneous group of diseases ranging from optic pits, segmental or global optic nerve hypoplasia to optic nerve aplasia, optic disc conformation anomalies and syndromes associating microphthalmia, colobomas, aniridia or brain anomalies (37–43). Single-case reports or small case series have identified a variety of genetic mutations linked to these anomalies and recent whole-genome-sequencing studies extend these lists considerably (44, 45). These findings can only be valued if mechanistic roles of these genes in the development of the optic nerve is demonstrated in animal models.

Similar to other white matter tracts in the brain, the optic nerve develops based on a few pioneer axons which use their growth cones to follow various guidance cues on their way to the targets (30). They are joined by the axons of the later-born RGCs by fasciculation (46). The first intermediate target for RGC axons is the optic nerve head (ONH), a region located at the junction between the future retina (the optic cup) and optic nerve (the optic stalk), resulting from complex morphogenetic movements of the optic vesicle – extensively reviewed (39, 40, 47–54). The necessity for the ONH in RGC axon development is demonstrated by the cases of retinal organoid cultures. In the absence of an optic stalk, retinal organoids are still differentiating and developing many anatomical and functional aspect of *in vivo* retinas, but are unable to grow RGC axons, as RGC survival is compromised (55, 56). This limitation was recently partly overcome by assembling retinal and thalamic organoids (57) or by culturing optic vesicle bearing brain organoids, that maintain the continuity between the optic vesicle and the brain (58). Identifying the ONH signals dedicated to RGC axon pathfinding is complicated by the coincident timing of morphogenesis of the optic cup and optic stalk, optic fissure closure and the escape of the first RGC axons out of the optic cup (59–61). An added challenge is to discriminate between primary RGC axon guidance defects and axon misrouting secondary to optic cup/stalk developmental anomalies such as coloboma or patterning defects (62, 63). The aim of this review is to survey the experimental results of the past decades on optic cup/optic stalk morphogenesis and early RGC axon guidance in conjunction with the recent RNA sequencing studies on developing retinas/RGCs in order to characterize the interplay between extracellular signaling molecules and intrinsic transcriptional pathways involved in the initiation of RGC axons pathfinding, which could be targeted in future retina/optic nerve regeneration strategies. The information presented in this review mostly comes from experiments done on mouse models. In case findings are coming from other species, the experimental models are mentioned in the text. We propose that the key to early RGC axon guidance is the spatial and chronological correlation between optic fissure closure and RGC differentiation initiation allowing the closing optic fissure to serve as a permissive channel for the pioneer axons, which are followed by the next axons by fasciculation.

## 2 Retina morphogenesis

**Figure 1:**
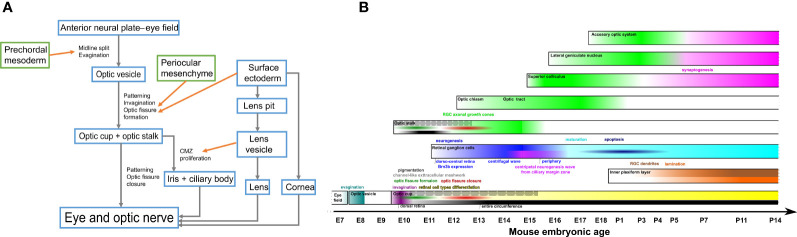
**(A)** Schematic illustration of eye morphogenesis. **(B)** Schematic developmental timeline of main events in mouse retinal ganglion cells development. grey arrow, developmental transformation; orange arrow, influence; RGC, retinal ganglion cells.

The first target of the RGC axons in their way to the retinorecipient nuclei in the brain is the optic disc. The position of the optic disc precursor region changes during the successive morphological rearrangements that take place during the morphogenesis of the eye (**Figure 1A**).

### 2.1 Eye field differentiation

The origin of the nervous system is the ectoderm, where a neuroectoderm is specified by BMP4 inhibition via follistatin, chordin and noggin (64). The anterior neuroectoderm is further induced by Wnt downregulation (64). As demonstrated in xenopus, within this region an eye field will be induced by signals coming from the adjacent mesenchymal tissues (65). Signals from the prechordal mesoderm including cyclops (Cyc), sonic hedgehog (Shh) and SIX3 split the eyefield in two (47, 49, 66). It further develops into two laterally placed optic vesicles under the influence of Eph/Ephrin signaling at an intersection between the Wnt and FGF pathways (40, 67).

### 2.2 Optic vesicle evagination

Lateral evagination of the forebrain precursor region leads for the formation of the optic pit that evolves to an optic vesicle (47, 68). The lateral expansion of the optic vesicles brings them in the vicinity of the lens-competent surface ectoderm. Close contact between the two structures is ensured by the displacement of the interposed mesenchyme and by a meshwork of collagen and cellular processes (47). The cavity of the optic vesicle is in direct communication with the ventricular cavity of the brain (69).

Optic vesicle formation occurs under the control of Rax, Pax6 and Tll (48). Activated by Sox2 and Otx from the anterior neuroectoderm, Rax represses NLCAM and induces CXCR4 acting on cell shape and movement with the important contribution of laminin (48, 70, 71).

### 2.3 Optic vesicle patterning

A recent single-cell RNA sequencing study (72) highlights the cellular heterogeneity of the optic vesicle comprising seven distinct neuroepithelial cell populations, four of which are stage-dependent presumptive retinal precursors. The optic vesicle becomes regionalized under the influence of eye field specific transcription factors including Lhx2, Pax6 and Six3, upregulated by Rax (48, 73). Anatomical orientation of optic vesicle patterning also relies on optic neuroepithelium cilia required for Hedgehog signaling, expressed in a proximal-high to distal-low gradient, as well as for PCP, Wnt, TGF-β, PDGFα, RTK, mTOR and Notch signal transduction (74).

The proximal domain of the optic vesicle will form the optic nerve whereas its distal domain will become the neural retina (NR) and retinal pigment epithelium (RPE) (75). These domains are initially delineated on a dorso-ventral axis so that the dorso-distal optic vesicle will form the future NR and RPE, and the proximo-ventral optic vesicle will become the ventral optic stalk (vOS) (47). The presumptive RPE and the ventral optic stalk each are continuous with the presumptive forebrain, but are separated from one another by the ventricular space (69). At this stage, RPE-NR and NR-vOS boundaries are fluid. Proximo-ventral fate is specified by hedgehog (Hh) through activation of Pax2, Vax1 and Vax2. In zebrafish, Hh also represses Pax6, the dorso-distal specifier, by expression of Mid1, a regulator of Pax6 ubiquitination (76). The NRE-vOS boundary is gradually sharpened also by Pax2-Pax6 mutual repression and Hes1 activity in the vOS (47, 77). Future ONH cells are tripotential and need Pax2 to shut off NRE and RPE fates, to adopt glial fate and to activate Hes1 (63). NR/RPE fates are specified by Vsx2 (or Chx10)/Mitf expression respectively, regulated by Lhx2 and lens-derived FGF signaling (48, 51). FGF soaked beads have the ability to convert RPE to neural retina in chicken (78, 79). In mice, the surface ectoderm provides FGF1 and 2 which activate VSX2, that in turn represses Mitf (80, 81). Among the multiple FGF ligands, FGF8 coming from the telencephalic vesicles has the main role in optic vesicle patterning, while the others are able to compensate in its absence, as shown in zebrafish (82). RPE differentiation requires the Wnt/beta-catenin pathway, including Porcn function (83).

### 2.4 Optic cup and optic stalk invagination – optic fissure

The optic vesicle undergoes a process of invagination between becoming a bi-layered optic cup (inner NR and outer RPE) and optic stalk in coordination with the invagination of the lens placode to a lens vesicle (69). The surface ectoderm covering the lens will become the cornea thus defining the mature appearance of the eye (47). The edge where the inner and outer layers meet plays a role in the invagination process and evolves into the ciliary body and the iris (40, 47, 84). Interactions between the optic vesicle and the surface ectoderm are essential for the invagination process (85) and are based on Pax6 regulated fibronectin 1 expression along with the retinoic acid (RA) signaling pathway (86, 87). RA local concentration is controlled by synthesizing (RALDH1/3) and catabolizing (CYP26A1/C1) enzymes and in chick retina it is localized complementary to FGF8 expression (88). Lhx2 is a regulator of both optic cup and lens formation (89). Optic cup derived RA also control the expression of periocular mesenchyme markers such as Pitx2 and FoxC1 (51).

Optic vesicle invagination is asymmetric (35, 90), more accelerated on the ventral side leading to the formation of a grove, the optic fissure (91) and to a reflection of the proximo-ventral/dorso-distal axis as the RPE enwraps the NR (47). The region where the proximal and distal portions of the optic fissure join will develop in to the optic nerve head (92). Formation of the optic fissure allows the mesenchymal cells to invade the optic cup and to form the hyaloid vasculature (93). While the vOS invaginates and forms the tissue through which the RGC axons will travel, the dorsal optic stalk will transform in non-neural tissue sheathing the optic nerve (47). The optic fissure domain is characterized by Netrin 1, Pax2, Vax1, Vax2 and Raldh3 expression (50, 93). Lower levels of Raldh3, Vax2 and Tbx5 and expansion of the Pax2 domain associated with increased apoptosis in the ventral retina was seen in Fz5 (a Wnt receptor) conditional knock-out mice (94). Optic fissure formation is induced by lens-independent signals including Pax2, Vax1, Vax2, Bmp7 (from the periocular mesenchyme), Shh and FGF (50, 91). Optic fissure formation is disturbed by experimental manipulations of these morphogens. Bmp7 knock-out mice have no optic fissure, Pax2 knock-out mice bear proximal optic fissure defects and RA induces optic fissure invagination in zebrafish (50, 91, 95).

As the optic cup grows, the optic fissure margins get closer to each other, displace the intertwining periocular mesenchyme and come in contact (51, 91). Optic fissure closure begins at midway and progresses both distally and proximally based on two distinct processes: fusion (basement membrane elimination) and intercalation (filling of the optic fissure space with newly differentiated astrocytes and incoming axons), which is more characteristic for the proximal part of the optic fissure (47, 51, 91). The hyaloid artery remains separated from the axons in the OS by a laminin cap contact (91). Optic fissure closure requires sharp delineation between the NR/RPE domains based on mutual restricting Mitf and Pax2 expression, regulated by Zfp503 (96) and FGF signaling via FGF receptors associated with Frs2α-Shp2 complex, ERK/Ras signaling (62, 97, 98) and Wnt-Fz5 signaling (94). The actual fusion process is promoted by TGFbeta (99). Netrin1 is directly involved in the fusion process in chicken (100).

As any morphogenetic movement based on proliferation and sculpting, optic cup invagination and optic fissure closure are accompanied by significant cell death. In mice, there is a sequential wave of cell death starting from the ventral optic cup, continuing along the fusing edges of the optic fissure and proceeding into the optic stalk followed by an invasion of macrophages from the surrounding mesenchyme that phagocytize the cell debris and are in close contact with the emerging RGC axons (101).

### 2.5 Optic cup and optic stalk patterning

Patterning in the optic cup and stalk follows the general domains established at the optic vesicle stage (102) and further compartmentalizes the structure along three axes (dorso-ventral, naso-temporal and proximo-distal) under the control of Hh signaling, as demonstrated in xenopus (103). The dorso-ventral patterning is achieved by dorsal Tbx5, Xbr1, COUPTFI/II and ventral Pax2, Vax2 expression (47, 69) as a result of Hh versus Bmp signaling, according to studies done in chick and frog embryos (65, 104). In zebrafish, nasal Foxg1 and temporal Foxd1 restriction is regulated by interaction between FGF and Hh signaling (105). EphA receptors and EfnA proteins are expressed in complementary nasal to temporal gradients, while EphBs/EfnBs have opposing dorso-ventral gradient expression in the NR (27, 106). Dorso-ventral patterning of the RPE is influenced by Zfp503 (96). Patterning along the third axis, the proximo-distal one, entails centro-peripheric regionalization in the optic cup, ONH delineation and OS-OC boundary delineation. The ONH domain expresses markers of the optic stalk (Pax2 and Vax1) and ventral neural retina (Netrin1, Vax2 and Raldh3) under the control of Bmp7 and Shh (93). The periphery of the OC is represented by the ciliary margin zone expressing Msx1 and Otx1 (107). The ciliary margin zone has a distal Bmp4 domain and a proximal CyclinD1/Msx domain containing multipotential retinal precursor cells (108). Optic cup periphery specification requires Wnt and Shh signaling, transduced via Cdon, Boc, Gas1 and Lrp2 (40). The outer and inner layers of the ciliary margins generate the outer and inner layers of the iris and cilliary body respectively, under Pax6 signaling (109). Sub-patterning of this region is based on FGF gradients interacting with Wnt signaling (98, 110). The NR/RPE boundary from the ciliary margin zone continues on the optic fissure margins (51).

The optic stalk also has two layers. In analogy to the RPE completely surrounding the neural retina as a result of invagination and optic fissure closure, the non-neuronal tissue derived from the dorsal OS is completely encasing the vOS derived Vax1 positive epithelium (111). The ventricular cavity of the brain is still continuous with the future subretinal space (112) as a narrow space separating the two layers in the optic stalk. As a directly visible mark of the ongoing patterning process at the optic cup-optic stalk boundary, melanin observable in the RPE as well as in the wall of the distal optic stalk, which are continuous, and is gradually eliminated from the optic stalk and restricted to the RPE (113). The transient optic stalk melanization is concomitant with the exit of the first RGC axons in the optic stalk, but pigmented or previously pigmented Pax2 negative optic stalk regions are avoided by nerve fibers (93, 113).The inner optic stalk Pax2 positive astrocyte precursor cells extend in the retina as a cuff that enwraps the exiting RGC axons, separating them from the subretinal space (68). There is a mutual influence between the RGC axons and these cells: on one hand, the Pax2 ONH cells provide axon guidance cues including Netrin1, NCAM or, laminin but on the other hand once ONH fate is induced by Bmp7 from the periocular mesenchyme, Shh secreted by the RGC axons is needed to maintain the ONH Pax2/Netrin1 cell population, which express Gli1 and Ptch Shh receptors (68, 93). In the absence of RGC secreted Shh, melanin, Pax6 and Mitf appear in the optic stalk (114). Transdifferentiation of optic stalk tissue to RPE was also seen in FGFr1/2 or heparin sulfate deficient mice (62, 115).

The developmental sequence of optic vesicle – optic cup and stalk morphogenesis and patterning ensures the anatomical continuity between the neural retina, the residence of RGC cell bodies, and the optic nerve precursor so that the RGC axons travel a natural course to the future optic chiasm region. As **Table 1** illustrates, any disruption in this sequence can disturb the early developmental steps of the RGCs.

**Table 1:** Mouse knock-out models for early retina morphogenesis developmental defects.

Genes of interest	Mutant mice	Neural retina	Optic fissure developed	Optic fissure closed	RGCs born	Axons emerged	All axons targeted OD	All axons remained in the ONFL	Optic Stalk developed normally	All axons exited the eye through the OD	Other changes
Bmp4	RaxCre; Bmp4 cKO (116)	Absent (RPE instead)	Not applicable	Not applicable	Not applicable	Not applicable	Not applicable	Not applicable	Not applicable	Not applicable	No lens induction
SoxC	Atoh7Cre; Sox4 cKO; Sox11 cKO (117)	Present	Identified	Fused margins	Nearly complete loss	Not applicable	Not applicable	Not applicable	No optic stak defects identified	ON aplasia	–
Bmp7	Bmp7–/– (93)	Present	Absent (stopped at optic vesicle stage)	Not applicable	Present	Intraretinal axons identified	OD absent	Axons gather either on the vitreal surface or in the subretinal space	absent OD	ON aplasia	Microphthalmia, absence of hyaloid artery
FGF	Six3Cre; Fgfr1cKOFgfr2cKO (62)	Present	Identified	Coloboma (Mitf was induced, Pax2 downregulated)	Present	Intraretinal axons identified	No misguided axons	Axons were misrouted in the sub-retinal space	Reduced in size, ectopic pigmentation	Thin or absent optic nerve	‘Tear-drop’- iris resembling uveal coloboma
Pax2	Pax2–/– (118)	Present	Identified	Coloboma	Present	Intraretinal axons identified	No misguided axons	All axons within ONFL	Extension of RPE into OS, all glial cells of OS absent	No misrouted axons identified	Chiasm agenesis, neural tube closure defects at hindbrain level
Shh	Thy1Cre; Shh -/cKO (68)	Present	Identified	Fused margins	Present	Intraretinal axons identified	Misguided axons identified	Axons enter the sub-retinal spaces in several regions of the retina and at the optic disc	Optic nerves were thin, hypocellular and surrounded by pigmented cells	Of the axons that arrived at the disc, some did not to exit and coiled in the sub-retinal space	Microcephaly, hypoplastic craniofacial structures, micropthalmia and failure of eyelid closure
Netrin	Netrin-1–/– (68)	Present	Identified	Margins in contact, but not fused completely	Present	Intraretinal axons identified	No misguided axons	Ectopic penetration through the full thickness of the peripheral retina	No optic stak defects identified	ON hypoplasia; once at the disc, many axons splayed out	–
Vax1	Vax1–/– (119)	Present	Identified	Coloboma	Present	Intraretinal axons identified	No misguided axons	All axons within ONFL	Aberrant RPE in OS	Axons restricted to a segment of OS away from glial precursors	Axons stall at the base of the hypothalamus, chiasm agenesis
Vax2	Vax1–/–; Vax2–/– (111)	Present (double volume)	Absent	Not applicable	Present	Intraretinal axons identified	Not applicable	Axons run in two parallel streams on the inner surface of duplicated RGC layer	OS replaced by retinal tissue extending to brain midline	Not applicable	RPE differentiation is limited, axons do not cross the midline, cleft palate

The table compares the effects of different gene knock-out manipulations in mice on the key developmental events involved in retina morphogenesis and early retinal ganglion cells (RGCs) axon guidance. cKO, conditional knock-out; RPE, retinal pigment epithelium; OD, optic disc; ONFL, optic nerve fiber layer; OS, optic stalk; ON, optic nerve.

## 3 Retinal ganglion cells development

### 3.1 RGC differentiation

Retinal precursor cells (RPCs) are able to generate all retinal neural cell classes and Müller glia, while astrocytes, macrophages and microglia later migrate into the retina (120). They commit to a specific fate as they transition from proliferative to terminal division states (121–123). The retinal cell types are produced in a stereotypic sequence, with RGCs, cones, horizontal and amacrine cells in a first wave and a second wave for bipolar, glial and a part of the amacrine cells, while rods differentiate throughout the retinal development time frame (120). RGCs differentiate in a central-to peripheral wave starting from the dorso-central retina, adjacent to the ONH (124, 125)

Uncommitted and lineage-restricted RPCs are located in the neuroblast layer, at the apical side of the NR, similar to the ventricular zone in the developing brain (126). Apolar RGC precursors become postmitotic in the neuroblast layer and become bipolar as their cell body translocates to the basal surface of the retina, where the ganglion cell layer will be located (122, 127). As they differentiate to RGCs, the apical process detaches and they become multipolar, growing an axon and dendrites (24). RGC precursors failing to differentiate undergo apoptosis in the ganglion cell layer (122). A subset of non-apoptotic new-born RGCs are eliminated 24h after birth by microglia based on complement signaling through phagoptosis (128).

A second source of retinal cells is the Msx1 precursor cell located at the ciliary margin zone (129). RGCs from the ciliary margin zone differentiate later than the central ones (31). Instead of translocating from the ventricular layer, they migrate laterally from the CyclinD1 zone directly in the ganglion cell layer (31, 108)

Still multipotential, RGC precursors are already committed to a specific type. The cell-type specification is continued in late embryonic and postnatal life through intrinsic transcriptional programs to reach the 40 types of mouse RGCs (26). For example, Ret-Brn3a interactions in postmitotic neurons can switch cell type/morphology (130).

### 3.2 RGC axon pathfinding

The vitreal process of the bipolar RGC precursors transforms into an axonal growth cone (24). RGC axons emerge very early during differentiation, even before the cell body has translocated to the ganglion cell layer (114) and start to express Gap43 and Tuj1 (131). RGC axons are already seen in the optic stalk coming from bipolar precursor cells with cell bodies at different hights in the retinal epithelium (132).

The most distal expansion of the axon is known as the growth cone (132), a sensory-motor structure capable of extending retracting processes called filopodia (thin) and lamellipodia (flat) in response to external signals (29, 133). Lamellipodia have a branched network of F-actin maintained by branching proteins such as Arp2/3 whereas in filopodia F-actin bundling proteins like alpha-actinin and fascin keep F-actin in parallel bundles (134, 135).

Growth cone steering (chemotropic turning) or growth cone collapse under the influence of axon guidance cues implies rapid changes in local protein levels achieved by local translation and protein ubiquitination (136, 137). Axon pathfinding is based on growth cone cytoskeletal reorganization, a sequence of F-actin addition on the plus-end of microtubules, retrograde F-actin flow and microtubule–F-actin coupling influenced by the strength of the adhesion on the substratum, as shown in aplysia ex vivo studies (138). Growth cones have a spread form and move fast on adhesive substrates and adopt contracted forms and stall on less adhesive substrates (132). Axon growth is an intermittent process, characterized by advances and pauses (139).

**Figure 2:**
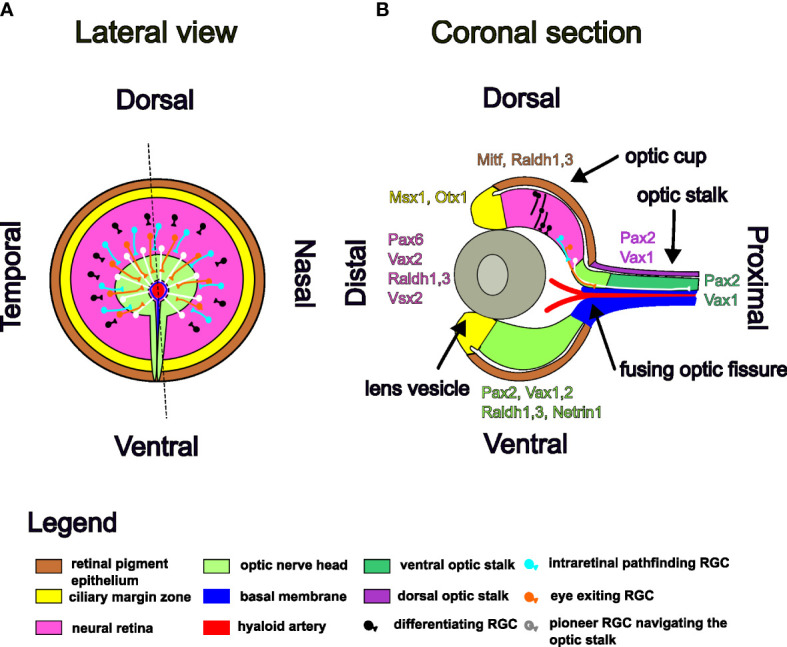
Schematic illustration of RGC axons pathfinding in the E12.5 mouse optic cup and stalk. Markers for each domain of the optic cup and stalk are listed in color code. **(A)** Lateral view. **(B)** Coronal section.

Once generated, RGC axons grow centripetally (**Figure 2**), within the optic nerve fiber layer and exit the eye through the ONH, enter the optic stalk within the neuro-epithelial lining of the optic fissure and travel along the optic stalk to the midline (24, 29, 140). Dye implant studies in rats and ferrets and mouse electron microscopy studies have shown that axon fibers do not preferentially occupy certain depths within optic nerve fiber layer or the optic stalk, newly added fibers being intermingled arbitrarly with the already present ones (112, 141, 142). In human fetuses, maturing and newly born axons are intermingled and the only ordering is at the entrance in the optic disc, where retina quadrant provenience is respected (143). This order of the axons at the ONH is lost within the optic nerve, so that axon guidance cues at the following checkpoints on their path to the targets are needed in order to ensure final retinotopic mapping (144). The next intermediate target is the optic chiasm, where the ipsi/contra-lateral projection decision is made. The axons continue their path in the optic tract and defasciculate at their final targets where they assume retinotopic positions according to their cell type and retinal eccentricities. These processes have been extensively studied and reviewed and are beyond the scope of this paper (29–31, 145).

### 3.3 Developmental timeline of mouse RGCs

The main events in the developmental timeline of mouse RGCs are summarized in **Table 2** and **Figure 1B**.

**Table 2:** Mouse retinal ganglion cell axon development timeline.

Day	Events
E7	Gastrulation Pax2 and Rax expression in the precursor fields of forebrain, ventral hypothalamus and eye. (118, 146, 147)
E8	Cephalic flexure Optic vesicle evagination (118, 146, 147)
E9	Neural tube closure Telencephalic and diencephalic vesicles get delineated. Optic vesicle and stalk are fully formed. Vax2 expression in ventral optic vesicle Rax expression in optic vesicle, optic stalk and ventral diencephalon. CD44/SSEA neurons are born (91, 118, 147–150)
E10	Optic vesicle invagination starts Opic fissure formation The retinal pigment epithelium is a 1–2 cell thick layer. Melanin is produced in the dorsal margin of the optic cup. The optic stalk starts thinning and elongating Rax is expressed in the entire retina. (91, 111, 113, 147–149)
E11	Opposite optic fissure margins come in contact Pax2 expression in the optic fissure region. Retinal precursor cells become postmitotic and translocate towards the vitreal side of the neural retina epithelium. First RGCs express Brn3b in the dorso-central retina, close to the optic fissure and extend the first axons which exit the eye. Shh expression in the optic chiasm precursor region junction splits in two. Pax2 expression extends in the gap so that it is continuous across the midline. (91, 118, 132, 141, 151–155)
E12	Optic fissure closure starts near the lens. The pigmentation of the outer retinal layer has progressed to the entire optic cup circumference. The optic nerve head starts to form. Shh is expressed in the RGC layer central retina, extending close to the edge of RGC differentiation. RGC Brn3a and Brn3c expression is initiated. Robo2 and slit1 are expressed in the dorso-central retina. Pioneering RGC axons reach the optic chiasm. There is no tight fasciculation. (23, 91, 118, 132, 148, 152, 154, 156–159)
E13	Optic fissure margins are fusing. Pigment is eliminated from the optic stalk. RGC axons are grouped in fascicles in the retina. Shh and Gli1 expression domain has extended to the periphery. RGC axons cross the midline. (24, 91, 113, 119, 120, 132, 149, 160)
E14	The neural retina is made up of two layers. RGC birth rate is at peak. The other neuronal cell types are beginning to be generated. Pax2 is confined to optic disc and optic stalk. EphB2, robo1, robo2, and slit2 are evenly expressed in the RGC layer. Slit1 expression has a ventral-high/dorsal-low gradient. Numerous RGC axons, coming from cells located in the central and midperipheral retina, have entered the optic tract. First RGC axons enter the superior colliculus Ipsilateral RGC axons reach the chiasm. (113, 124, 140, 141, 154, 157, 159–163)
E15	EphB2 gradient in outer retina. Growth cones are less numerous in the optic nerve compared to previous ages, indicating that the majority of the RGC axons have already passed this region at this time. Most RGC axons enter the superior colliculus. (23, 132, 161)
E16	Hyaloid vessels are ensheeted in a laminin cell cap in the optic nerve Astrocyte precursors appear in the optic stalk. The distribution of ipsilaterally projecting RGCs in the retina is delineated. EphB2 gradient in outer and inner retina. No growth cones in the optic nerve. Optic tract reaches its targets. Crossed accessory optic tract is formed. Ipsilateral fibers are seen in the geniculate bodies region, but not at the superior colliculus. (91, 124, 132, 140, 161, 164)
E17	Ipsilateral RGC differentiation is finished. The number of RGC axons in the optic nerve is maximal and begins to decrease. Robo1 and slit 2 are restricted to the inner retina. Slit 1 is absent. (141, 159)
E18	Medial terminal nucleus, dorsal and ventral lateral geniculate nuclei are innervated by RGC axons. (140)
P1	Dorsal and ventral RGC axons are separated on the optic tract, while nasal and temporal axons are intermingled. (144, 165–167)
P3	RGCs are growing dendrites. RGC axons form synapses in the target nuclei. (122, 168)
P4	Inner plexiform layer lamination is forming. Superior colliculus projections are complete. Lateral geniculate nucleus projections are sparse. (169, 170)
P5	The optic nerve contains oligodendrocytes and immature astrocytes (164).
P7	RGC dendritic arbors maturation is nearly complete (168, 171, 172)
P11	Inner plexiform layer lamination is defined. (169)
P14	Superior colliculus and lateral geniculate axonal arbors are mature. (170)

The table summarizes experimental findings in wild type mice from multiple studies exploring the developmental stages of retinal ganglion cell (RGC) axons, grouped according to the gestational or postnatal age, which is indicated by the embryonic/postnatal day of life.

## 4 Transcriptional profiles of early mouse RGCs

The behavior of RGC axons in Atoh7−/−and Atoh7−/−;Bax−/− mice, growing in the nerve fiber layer but failing to exit an apparently normal ONH, suggests that intrinsic RGC transcription programs are required for eye exit in addition to ONH guidance (173). Brn3b and Isl1 ectopic expression from the Atoh7 locus in Atoh7 knock-out mouse retinas rescues the axon guidance phenotype (174) showing that Atoh7 is indirectly involved in axon guidance by inducing Brn3b and Isl1. There is very little knowledge on transcription factors and downstream genes involved in RGC axon guidance, and identified phenotypes involve events that occur later than eye exit. Delayed axon growth and abnormal axon de-fasciculation from the optic tracts was seen in Brn3b knock-out mice (23, 169) and ipsi-/contra-lateral projection phenotypes were observed in Zic2, Isl2 and Sox4,11,12 mutants (117, 175–181).

RGCs are the first differentiated cells in the neural retina and axon emergence and pathfinding is the major developmental process they are involved in. In this context, RNA sequencing studies of newly born RGCs have the potential to identify the transcriptional pathways involved in pioneer axon pathfinding. However, due to the technical challenges such as the small size of the retina and the small cell number there are only a few published papers more or less directly focused on newly born RGCs (21, 26, 168, 173, 182–186). According to Shekhar et al. (26), there is a good overlap between their developing retina single cell RNA sequencing data and the other two studies using the same methodology, namely Clark et al., (184) and Giudice et al., (182).

**Table 3** presents a selection of genes resulted from three RNA sequencing studies using different approaches: the first study (168) used immunomagnetic sorting of dissociated E15 retinas to sequence RGC RNA against retina supernatant, the second study (182) performed single-cell RNA sequencing on E15 retinas and identified newly born RGCs by unbiased clustering, and a third study (183) performed bulk RNA sequencing on E11 to P28 retinas. The genes were also looked up in microarray studies in embryonic retinas of Atoh7 knock-out mice (185, 186), RNA sequencing in isolated embryonic RGC growth cones (137) and public *in situ* hybridization databases (Allen Brain Institute and Eurexpress). The selection resulted from the logical intersections between lists of genes identified in relevant categories of samples in the three studies (for complete lists and intersection strategies see **Supplementary Table 1**). Cellular localization of the genes according to https://www.ncbi.nlm.nih.gov/gene/ is presented in **Supplementary Table 2**. Our selection included some proteins belonging to the Netrin1-Dcc signal transduction pathway, namely App (Stmn2, Kif1b), Cdc42, Trim67, Tubb3 (187–191). Manipulations of some of the identified genes/proteins produce RGC axon guidance errors: Cntn2 deficiency is linked with axon fasciculation and contralateral projection defects (192); Dcc knock-out results in failure of RGC axons to exit the eye (151); Gap43 null RGC axons have chiasm crossing defects (193); Igf1 and Igfbpl1 contribute to RGC axon growth by intracellular Calcium level modulation and mTOR pathway activation (194); antibodies against Nfasc induce de-fasciculation in chick RGC cultures (195); Nrcam is required for chiasm crossing (196); Nrp1 conditional knock-out causes chiasm crossing and optic tract fasciculation defects (197) and Tenm3 deficient RGC axons fail to project ipsi-laterally (198, 199). Others genes in the list - App, Cdc42, Celsr3, Chl1, Elavl4, Evl, Islr2, Kif1b, Kit, Kitl, Mmp24, Stmn2, Tubb3 - are associated with axon growth or guidance defects in other regions of the nervous system or in cultured neurons (191, 200–213).

**Table 3:** Genes expressed in developing retinal ganglion cells (RGCs).

Gene name	Giudice 2019 E15.5 (182)	Sajgo, 2017 E15 RGCs (168)	Brooks 2019 Retina (183)	Gao, 2014 E13.5 Atoh7 dependent (185)	Mu, 2005 Atoh7 dependent (186)	Zivraj, 2010 E16 cultured RGC growth cones (137)	Allen Brain Institute Mouse ISH Data Atlas	Eurexpress Mouse ISH Data Atlas
App	young RGCs	x					RGCs e11.5<e13.5<e15.5	e14.5 RGCs
Cdc42	young RGCs	x					weak	e14.5 RGCs
Celsr3		x	e12				RGCs e11.5<e13.5>e15.5	e14.5 RGCs
Chl1	old RGCs	x	e14			x	RGCs e11.5<e13.5<e15.5	
Cntn2	old&young RGCs	x	e12&e14	x	e13.5<e14.5>e16.5>e18.5	x	RGCs e11.5<e13.5<e15.5	e14.5 RGCs
Dcc	old&young RGCs	x	e12&e14	x			RGCs e11.5<e13.5>e15.5	e14.5 RGCs
Elavl4	young RGCs	x	e12&e14	x	e13.5>e14.5>e16.5>e18.5			
Evl	young RGCs	x					RGCs e13.5>e15.5	
Gap43	old&young RGCs	x	e14	x	e13.5>e14.5>e16.5>e18.5	x	RGCs e11.5(central)<e13.5(central)<e15.5(all)	e14.5 RGCs
Igf1	old RGCs	x					RGCs e15.5	e14.5 RGCs
Igfbpl1	young RGCs	x	e12&e14	x	e13.5<e14.5>e16.5>e18.5		e11.5 weak	e14.5 RGCs
Islr2	old&young RGCs	x	e14	x				e14.5 RGCs
Kif1b	old RGCs	x						e14.5 RGCs
Kit	young RGCs						e11.5<e13.5	e14.5 RGCs
Kitl	old RGCs	x					RGCs e11.5<e13.5>e15.5	e14.5 RGCs enriched
Mmp24		x	e12&e14	x				e14.5 RGCs
Nfasc	young RGCs	x	e12&e14				RGCs e13.5>e15.5	e14.5 RGCs
Nrcam	young RGCs	x	e14				RGCs e13.5<e15.5	
Nrn1	young RGCs	x	e12&e14	x			RGCs e13.5<e15.5	
Nrp1	young RGCs	x	e14	x			RGCs e13.5>e15.5	e14.5 RGCs
Stmn2	young RGCs		e12&e14		e13.5>e14.5>e16.5>e18.5	x		e14.5 RGCs
Syt13	young RGCs	x	e12&e14	x	e13.5<e14.5>e16.5>e18.5			e14.5 RGCs
Tenm3	old&young RGCs	x					RGCs ventral e11.5>e13.5>e15.5	
Trim67		x	e12&e14	x		x		
Tubb3		x	e12&e14	x	e13.5>e14.5>e16.5>e18.5		RGCs e11.5<e13.5>e15.5	e14.5 RGCs

“x” denotes that the gene was found to be expressed over the threshold by the study referred to in the column. In studies including specimens of multiple embryonic ages, the ages where gene expression was found are indicated by the day of embryonic life. The expression level differences among embryonic ages are indicated by “>“/”<“ signs. The last two columns represent in situ hybridization (ISH) studies and they include the embryonic age and the cells where gene expression was found.

## 5 Signaling – transcription interactions in early axon pathfinding in mouse RGCs

### 5.1 General principles

**Figure 3:**
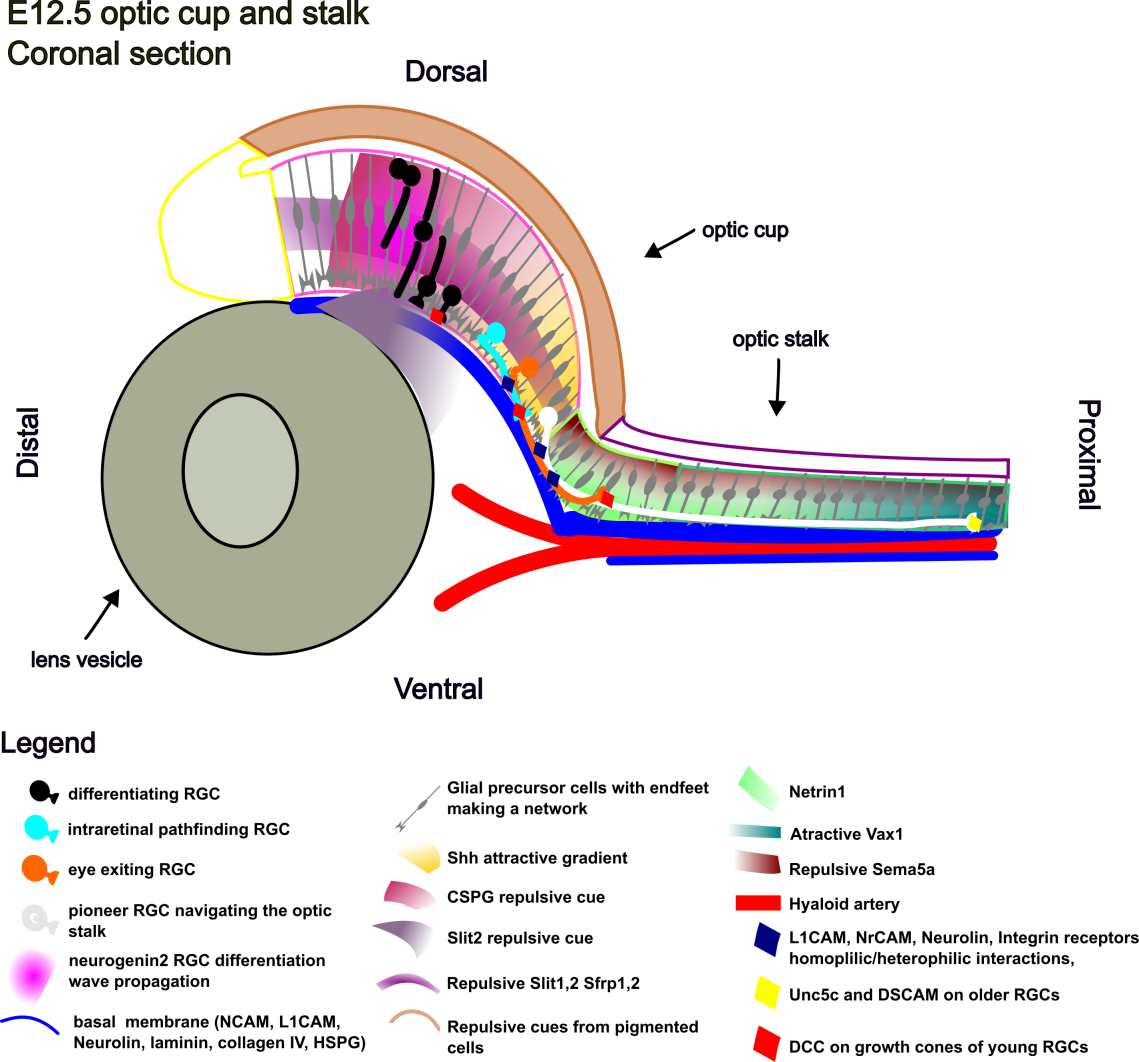
Schematic illustration of RGC axon guidance cues in the E12.5 mouse optic cup and stalk.

RGC axon pathfinding implies pioneer axon guidance and later born axons fasciculation (214). Pioneer axons navigate in the retina based on chemotaxis (attractive and repulsive cues forming gradients) and haptotaxis (physical interactions with permissive substrates) (30, 91, 215, 216). The next paragraphs survey the evidence on the regulation of RGC differentiation timing, haptotaxis and chemotaxis conditions for the pioneer RGC axon guidance and on RGC transcriptional programs involved in pioneer axon pathfinding and cofasciculation (**Figure 3**). The molecular determinats of retina development known from mouse studies and their corresponding human phenotypes are listed in **Supplementary Table 3**.

### 5.2 Regulation of RGC differentiation

Retinal cell type differentiation sequence is a result of RPC intrinsic programs and extrinsic cues (48). RPC proliferation and multipotential state is regulated by Vsx2, Pax6, Six3, Six6, and Sox2 (126). Notch and Shh signaling keep RPCs in the proliferative state (217). Notch-Delta signaling maintains the progenitor pool by lateral inhibition (49, 218) and contributes to the transition from naïve to competent RPCs (145). When Delta-Serrate-LAG2 ligands from adjacent cells bind to the Notch extracellular domain, the intracellular domain together with RBPJ and MAML1 translocates in the nucleus and activates Hes1 and Hes5 transcription. When ligands and receptors are expressed by the same cell, the Notch pathway is inhibited. Notch expression is activated by Sox2 and suppressed by cell type specific factors like Atoh7, Ascl1, Ptf1a, and Foxn4 (175, 219). Sfrp1/2 also deactivate Notch signaling via Adam10 (220). miRNAs maintain the RPC competence window for RGC differentiation (221, 222).

Experiments in zebrafish and chicken have shown that Shh signaling from the midline and FGF signaling from the OS trigger RGC differentiation (223, 224). OS derived FGF3 and FGF8 initiate neurogenesis in the central retina (78, 224). FGF8 is negatively regulated by retinoic acid (88). Retinoic acid catabolizing enzymes Cyp26a1 and Cyp26c1 are expressed in an equatorial streak (225) characterized by higher RGC density (88). Conditional FGFR1/2 double knock-out in mouse RPCs impairs RGC differentiation onset (97). The FGF–Frs2–Shp2 pathway controls RPC proliferation (62, 97, 115). Ikaros is involved in the production of early RGCs (121, 226).

Once started by FGF signaling, the RGC genesis wave progresses to the periphery based on Shh signaling, as observed in zebrafish (227). Brn3b regulated (168) secretion of Shh from RGCs modulates proliferation and differentiation of RPCs (68) and is required for the maintenance of the RPC pool (120). Neurogenin2 and Ascl1 are also responsible for the propagation of the RGC genesis wave (175). Neurogenin2 is expressed ahead of the RGC wave edge and regulates Atoh7 transcription in RPCs (228). Secondary RGC genesis from the ciliary margin zone is regulated by CyclinD2, a cell cycle facilitator (31, 108).

Pax6 activates transcription factors that commit RPC to different fates so that in absence of Pax6 only amacrine cells are produced (126). Downstream of Pax6, two proneural transcription factors are Neurog2 and Atoh7, which is also under the control of Gdf11 and follistatin (49). Atoh7 expression in RPCs determines competence acquisition, not RGC fate commitment and its absence nearly eliminates RGCs (229). Neurog2 and Atoh7 activate RGC specification transcription factors including Sox4, Sox11, Neurod1, Brn3b and Isl1 (230). The Atoh7-Brn3b pathway suppresses non-RGC transcriptional programs and accounts for 70% of RGC differentiation (123). Brn3b further activates Brn3a, Brn3c, Eomesodermin, Ebfs, Onecut1, and Onecut2. Isl1 is required for RGC specification having overlapping targets with Brn3b (168, 231). Ectopic expression of Brn3b and Isl1 from the Atoh7 locus in Atoh7 knock-out mouse retinas rescues RGC differentiation (174). Other than Brn3b and Isl1, NeuroD1 or SoxC can also partly compensate for the absence of Atoh7 (175, 232). Dlx1/2 are expressed at transition stages of RGC fate commitment, and are negatively regulated by Brn3b and Isl1, and the Bmp and Vegf pathways also contribute to RGC differentiation (175, 233).

### 5.3 Regulation of RGC axonogenesis

The axons grow directly from the basal aspect of the RGCs concomitant with apical process detachment (127). Polarized organization of cytoskeletal structures governed by instrinsic mechanisms was identified in various neuronal populations prior to axon emergence (27, 234). RGC axon sprouting is controlled by integrins and cadherins (22). FGFs stimulate axon generation and growth in xenopus RGC cultures (235). Experiments in mouse cortex have shown that apically oriented axon genesis is linked with the movement of the centrosome apical to the nucleus and that this polarization is regulated by TGF-beta – LKB1 –BDNF signaling (127, 236). The orientation is reversed in the retinal neuroepithelium, which has a basal lamina made of laminin, collagen IV, nidogen, agrin, condroitin sulfate proteoglycan (CSPG) and heparan sulfate proteoglycan (HSPG) (114). Laminin contact directly promotes axon sprouting by stimulating the accumulation of Kifc560, an early axonal marker, and the formation of growth cones (127). Glial polarity precedes neuronal polarity and studies in chicken retina explants have shown that glial endfeet promote axon formation while glial somata support dendritic growth (215). As the axon grows, the proximal segment loses the filopodia and takes a cylindrical shape and the ventricular process completely disappears (24). In zebrafish, apical retraction requires Slit1b-Robo3 signaling (237). Dominant negative N-cadherin expression leads to premature detachment in zebrafish (237) and blocks RGC axonogenesis in xenopus (18). Brn3b and Brn3c activate genes involved in axon formation and in their absence RGC neurites adopt dendrite-like features (152).

### 5.4 Regulation of growth cone dynamics

Growth cone steering and axon growth imply cytoskeleton reorganization which is mainly triggered by cell adhesion molecules (CAMs) (238, 239). Microtubule dynamic is modulated by several signal transduction pathways mostly based on kinases (240). Immunoglobulin superfamily CAMs involved (L1CAM, NCAM1, ALCAM, and CNTN2) activate Erk MAP kinase to promote axon growth in fasciculation because they are only expressed on axons, and not on the other substrates (241). RGC axons grow preferentially on L1CAM compared to extracellular matrix proteins such as laminin (242). Anti-L1 Fab and anti-NCAM Fab treatment had different effects on RGC axon growth cones in culture: direction change and lower growth speed versus increased elongation speed and premature growth stop respectively (243). FGF receptor mediated activation of the phospholipase C gamma cascade is needed for RGC axon growth in response to L1CAM in mice (242, 244). FGF signaling is also transduced by the Ras/MAPK and PI3K pathways (40, 115).

Cadherins are adhesion molecules expressed in the retina that promote axon growth by homophilic interactions. N-cadherin may play a dual role: it promotes neurite extension by sequestrating beta-catenin, and preventing the inhibition of adenomatous polyposis coli (APC) protein, a positive regulator of neurite growth. On the other hand, by binding to the cytoplasmic p120 catenin N-cadherin prevents GTPases Cdc42 and Rac1 from actin remodeling and thus has a growth inhibitory effect that prevents excessive axon growth at specific locations (245). In rats, transmembranar cadherins Celsr2 and Celsr3 have opposite effects on neuron-neuron contact triggered neurite extension based on homophilic interactions and downstream CAMKII (calcium/calmodulin-dependent protein kinase II) or calcineurin induction (246).

Studies in xenopus have revealed that in response to external cues such as a Netrin1 gradient, asymmetric cap-dependent translation of beta-actin is activated via phosphorylation of the translation initiation factor 4EBP, resulting in the pronounced extension of the filopodia located in the part of the growth cone exposed to the highest Netrin1 concentration (247).

### 5.5 Optic disc directionality

At the time of axon emergence, RGCs extend multiple transient minor processes to probe the environment for guidance cues and the ones oriented towards the attractive and away from the repellant cues will develop into the single axon, directed to the optic disc (243).

#### 5.5.1 Attractive cues

The ONH domain exerts attraction on the RGC axons as illustrated by the misrouting of RGC axons towards the margins of the unclosed optic fissure, expressing ONH markers, in Fz5 conditional knock-out mice (94). Netrin-1 on the processes of optic nerve head glial precursor cells is acting as a chemotactic attractant for the axons expressing its canonical Dcc receptor (238, 248). DCC is preferentially expressed by the newly born RGCs that are sending their axons to the optic disc (182). A central-high/periphery-low gradient of Shh is also an attractive guidance cue acting on Ptc-Smoothened, Hedgehog interacting protein (HiP) and Boc receptors expressed by the RGCs (92, 249). RGCs themselves are a source of Shh having a dual role in axon guidance and glial cell development (68). Blocking the FGF receptor or the signal transduction pathway in rat retina explant cultures causes new RGC axons to lose the optic disc directionality and to grow towards the periphery (242).

#### 5.5.2 Repulsive cues

The expression of the repulsive cues is complementary to that of the attractive cues, namely a periphery-high/central-low gradient (29). They are either secreted by the lens like Slit2 (250), or they are produced in the basal lamina in a wave preceeding the peripheral side of the newly born RGCs as it is the case for chondroitin sulphate proteoglycan (CSPG) (243, 251). Repulsive cues are regulated by transcription factor Zic3, with a periphery-high to central-low gradient of expression (114).

### 5.6 Optic nerve fiber layer restriction

#### 5.6.1 Physical substrate

RGC axons grow in a narrow space delineated by RGC cell bodies and the vitreal basal lamina (the inner limiting membrane) (35). This space is occupied by the endfeet of glial precursor cells, similar to the radial glia in the brain, which are organized in a channel-like structures forming a network that orients the emerging axons (216). In chicken retina cryocultures, axons preferentially follow glial precursors endfeet compared to preexisting axons or laminin (215).

#### 5.6.2 Attractive cues

Contact with glial precursors endfeet and the basal lamina is maintained on the basis of cell adhesion molecules such as NCAM and L1CAM as well as extracellular matrix proteins including Neurolin/DM-GRASP/BEN and NrCAM (29). In chicken retina, growth cones respond to a CRYPa1 receptor ligand expressed on the glial precursors endfeet by activation of rac and rho via the Trio protein resulting in axon growth and maintained contact between the RGC lamellipodia and the basal membrane (252). Basal membrane laminin binds to integrin receptors on growth cones and activate Rac and Cdc42 to promote axon extension (252).

#### 5.6.3 Repulsive cues

RGC growth cones are prevented from entering the deeper layers of the retina by neuroepithelial precursor cells somas, which have a repulsive effect on RGC axons but are permissive for RGC dendrites in cryoculture experiments (35, 253). Slit1 and Slit2 from the RGC and inner nuclear layers also repel Robo2 expressing RGC axons and their absence causes RGC axon misrouting in the outer retinal layers (29, 254–256) . RGC axon fasciculation defects within the optic nerve fiber layer, together with invasion of the INL, ONL and subretinal space are also seen in mice missing both Sfrp1 and Sfrp2 (257) and repulsive signals from pigmented cells in the outer retina keep the RGC axons from entering the subretinal space (113, 118).

### 5.7 Intraretinal fasciculation

Pioneer RGC axons serve as guides for the newly born axons so that optic disc targeting and nerve fiber layer restriction are achieved by fasciculation. Transient minor processes of newly born RGCs contact axons of more mature RGCs (243) and form bundles mainly based on immunoglobulin superfamily CAMs trans-homophilic interactions (27, 46). In goldfish, such molecules include L1, NrCAM or neurolin (258). In addition to hemophilic interactions, L1CAM also has heterophilic interactions with integrin receptors (259). FGF receptor blocking causes de-fasciculation in rat embryonic retina explant cultures (242). Transcription factor Irx4 has been shown to play a role in RGC axon fasciculation by down-regulating Slit1 (260). Inhibitory EphB proteins contribute to fasciculation in the dorsal retina (261). Several receptor-ligand pairs have been found to be complementary expressed in newly born versus maturing RGC and assumed to contribute to fasciculation (182).

### 5.8 Entering optic stalk/exiting the optic cup

#### 5.8.1 Physical substrate

After reaching the ONH region RGC axons pause, make a 90 degrees turn and exit the eye into the OS (262). The optic fissure margins are in contact and the fusion process is ongoing when the first RGC axons are exiting the eye (91). The presence of the optic fissure is essential for RGC axon exit, as demonstrated by the aberrant projection of axons in the vitreous or in the subretinal space leading to optic nerve aplasia in Bmp7 knock-out mice lacking an optic fissure and hyaloid artery (68, 93, 114, 263).

The path of the axons is not in the fissures’ lumen, which is occupied by the hyaloid artery, but within the neuroepithelial cells forming its walls (216). Axons are separated from the hyaloid artery by a laminin sheet (91). In continuity with their retinal homologues, optic stalk glial precursor cells have processes that form channel-like networks enclosing the axons (151). The timing of appearance and propagation of this meshwork of cellular processes is correlated and preceding the wave of RGC differentiation (216).

A potential physical substrate in the optic stalk is represented by the rare retinopetal fibers coming from the diencephalon (264). In the ferret, these fibers are transient and occupy the optic stalk before the entrance of the pioneer retinofugal axons (265).

#### 5.8.2 Attractive cues

The formation of the channel-like extracellular spaces is accompanied by cell death (216). NGF secreting macrophages invade the central retina and optic stalk shortly before RGC axon emergence to clear cell debris resulted from the apoptosis related to optic cup morphogenesis (101). The NGF receptors TrkA and p75^NTR^ are expressed by RGC axons at this developmental stage (101, 266).

For the molecules expressed in the ONH region it is difficult to distinguish their role as OC-OS morphogens from the role as axon guidance cues (114). ONH Pax2 positive cells form a cuff that guides the axons to the OS keeping them isolated from the RPE domain (153). They extend processes expressing Netrin1, an attractive cue acting on Dcc (93). Mutant mice deficient for Netrin1 or Dcc have optic nerve hypoplasia due to the inability of RGC axons to leave the eye in spite of having arrived at the ONH (248). R-cadherin is also an attractive molecule expressed by the ONH cells in chicken (267). ONH cells identity and function are under the control of Pax2, Vax1 and Vax2 (93). Shh secreted by early-born RGCs is also involved in the development of the ONH Pax2 positive cells, so that its conditional deletion from RGCs in ThyCre Shh null/floxed mice is associated with reduced number of axons exiting the eye and misrouting in the subretinal space (68). The fact that a good number of axons are still exiting the eye in these mice may indicate that to a certain extent pioneer RGC axons are able to exit the eye without attractive cues, only based on optic fissure vicinity (optic fissure formation is Bmp7 dependent, unaffected in these mice). As RGCs do not secrete Shh, the differentiation of the ONH cells is impaired and they do not provide attractive cues for the later born RGC axons, maybe counting for the misrouting observed. From this we can infer that pioneer RGC axons enter the optic stalk physically guided by the closing optic fissure and secrete Shh to make the ONH produce attractive cues for the later born RGCs.

#### 5.8.3 Repulsive cues

The change in growth direction at the ONH requires reverse signaling from attraction to repulsion so that axons growing towards the optic disc do not pass over it attracted by the cues on its opposite edge, but stop and enter through its center in the optic stalk. Fasciculation on other L1CAM expressing RGC axons coming from the opposite side of the retina must also be avoided otherwise axons are misrouted from one half of the retina to the other (27). Expression of inhibitory molecules such as EphA4, EphBs, Bmpr1b and NrCAM counteracts excessive fasciculation or axon stray in the subretinal space (29, 268).

The response of RGC axons to Netrin1 can be reversed based on the concomitant signals that regulate the intracellular level of cAMP or on expression of different Netrin1 receptors (187, 269). Laminin1, abundant in the basal lamina of the retinal vitreal side and closing optic fissure margins (91), binds to beta1 integrin receptors and blocks the cAMP increase induced by Netrin1 in RGCs thus changing Netrin1 attraction to repulsion (32, 262). By this mechanism, axons are guided away from the vitreal cavity and the optic fissure lumen and towards the intercellular spaces of the optic stalk neuroepithelium where laminin is absent and Netrin1 maintains its attractive effect (**Figure 3**, neuron represented in orange).

### 5.9 Traveling along the optic stalk

#### 5.9.1 Physical substrate

In the optic stalk, RGC axons grow mostly in the ventral part, between non-neuronal cells with processes enwrapping the axon bundles (256). These cells are differentiated from vOS precursors or migrated from the diencephalon, as is the case for oligodendrocytes (118). Small separated bundles travelling between the neuroepithelial cells as well as independent growth cones are seen at the early stages, whereas later the optic stalk is occupied by compact axon fascicles with intermingled astrocyte precursor cells (112, 270).

#### 5.9.2 Attractive cues

Later born axons fasciculate on the more mature ones in tight bundles (256) based on homophilic L1CAM interaction (243). Vax1 expression in the optic stalk and ventral diencephalon promotes growth cone progression from the ONH to the chiasm region (30). Transient retino-retinal projections were identified in multiple species including mice, projecting to the nasal retina (271). These misrouted axons probably come from the chiasm region and wrongly fasciculate with the fibers from the contralateral optic stalk.

#### 5.9.3 Repulsive cues

While traveling through the optic stalk, RGC axons have to be prevented from straying away in the surrounding tissues. Netrin1 expression extends from the ONH cells to the OS neuroepithelium displaced peripherally by the incoming nerve fibers and has a repulsive effect thus keeping the axons in the center of the OS (248). Growth promoting Dcc receptor expression in newly born RGCs is switched to Unc5c and DsCAM on the maturing RGCs (whose axons are already in the optic stalk), receptors that respond to Netrin1 signals by inducing growth cone collapse (182, 269). Another barrier is a ring of Sema5a expression at the basal side of the optic stalk neuro-epithelial cells with axon growth inhibition and growth cone collapse effect (272, 273). Repulsive Slit2 expression is also detectable in the OS at the time it is invaded by RGC axons and is thought to contribute to their restriction to the ventral side of the OS (256, 273).

## 6 Discussion

This review harmonizes recent findings with classic studies on optic cup and stalk morphogenesis and early RGC axon guidance. We summarized the key molecular determinants of the two processes, as proven by genetic, immunological or pharmacological manipulations in animal models and we also extracted a list of genes expressed in RGCs during the developmental period of early axon path finding, whose functions in this process remains to be explored in future studies. The reviewed data orients our current understanding of this developmental event towards new directions which will be exposed here along with some unanswered questions that we propose for this research field.

As it was described in the first paragraphs of this review, morphogenetic movements bring the precursor tissue of the optic nerve head from the ventral diencephalic midline region to its final position, in the center of the neural retina. Such movements have been documented by software based cell tracking in zebrafish embryos (274). The RGC pioneer axons will follow almost the same path, but in the opposite sense, on the way to their next target, the optic chiasm. Anatomical continuity on this path is maintained throughout the morphogenesis of the optic cup and stalk by means of the optic fissure formation. By the time the pioneer RGC axons approach the optic stalk entrance, the fissure margins grow towards each-other, come in contact and begin to fuse. There is a narrow slit left at the OC-OS junction which guides the pioneer axons into the optic stalk. OS cells have apical – basal polarity, such that certain permissive cues are present on the lumen side, while repulsive cues are sequestered in the lateral walls of the epithelium, restricting penetration (273). Pioneer RGCs differentiate in the dorso-central retina next to the ONH precursor domain. The spatial and temporal correlation between ONH morphogenesis and pioneer RGCs differentiation appears to be essential for the correct pathfinding of the RGC axons.

Common trigger signals for the two events are yet to be identified. As a first evidence, FGF-RA signaling seems to play a key role in linking optic cup/optic stalk boundary delineation and optic fissure formation and closure to RGC differentiation (62, 78, 88, 97, 242). However, future work is needed in order to establish whether RGC axon misrouting caused by disruption of FGF-RA signaling is the result of ONH development anomalies or of intrinsic RGC developmental defects.

The interaction between the developing ONH and the emerging RGC axons is bidirectional. In one direction, the ONH guides RGC axons based on chemotaxis and haptotaxis. Early born RGCs are able to respond to the signals coming from the ONH by expressing cell surface receptors and by activating transcription programs that promote axon growth and axon steering events. Axons of RGCs from Atoh7 null mice, kept alive by Bax knock-out, are unable to target the ONH and to exit the eye in spite of receiving the correct signals from the target (173). Recently, adult mouse Mueller glia were reprogrammed to neurogenic state *in vitro* by virus mediated expression of Atoh7 (275) and *in vivo* conditional expression of Brn3b, Islet1, Ascl1 and Atoh1 resulted in RGC-like neurons with morphological and electrophysiological RGC properties, which did not send axons to the ONH in spite of expressing many axon growth and guidance promoting genes (276). A potential future direction of research would be to also reprogram optic nerve astrocytes to secrete axon guidance molecules for the new RGC axons or to engineer the new RGCs to penetrate the adult lamina cribrosa, by uncovering and neutralizing the inhibitory cues. Another direction could be to simulate the physical properties of the closing optic fissure in order to promote haptotaxis based axon guidance. Experiments with spheroids of human stem cell-derived motor neurons showed their capacity to spontaneously assemble into an unidirectional fascicle when cultured next to a narrow channel (277). Simulating developing ONH environment locally may be also a solution for promoting RGC axonogenesis and survival in retinal organoids. Later born axons find their way to the ONH based on fasciculation on pioneer axons. In zebrafish retina, late born RGC axons cannot target the OD in the absence of early RGCs although the OD cues are present (278). This observation has two possible explanations: the canonical one is that pioneer axons actively find the ONH region and later born RGCs are guided passively so that they are not able to actively respond to ONH attractive cues but an alternative possibility would be that both pioneer and late born axons are guided passively to the ONH and only the position of the first RGCs next to the closing optic fissure enables them to exit the eye. The approaching margins of the optic fissure bringing the first axons in contact so that they can fasciculate may be enough for their growth out of the eye. For the RGC axons arriving at the optic disc after the optic fissure closed there is no physical path out of the eye, so that chemotaxis and fasciculation are become essential for axon guidance. Live imaging studies capturing the behavior of these later born axons at the optic disc are needed to confirm this hypothesis. Further studies involving desynchronizing optic fissure fusion and initiation of RGC differentiation or de-localizing the initial RGC differentiation spot could verify this later hypothesis.

In the other direction, RGCs also influence the ONH development. The reviewed studies on Shh secretion by RGCs and its role in ONH cells development as well as retinal precursor cell modulation indicate that RGCs actively influence the development of their path to the brain as well as the surrounding retina. More work in this direction should be done in order to find all the secreted molecules involved in this processes and their potential application in RGC regeneration strategies.

In summary, RGC axons follow a centripetal course within the inner most layer of the retina towards the optic disc and enter the optic stalk. In spite of the appearance of the mature optic nerve, RGC axons are not piercing through the wall of the eye, but they are gliding on a continuous path that is created during the morphogenesis of the optic nerve. They are guided by chemotaxis and haptotaxis cues provided by the developing optic cup and stalk and by fasciculation with their more mature neighbors. A profound understanding of the developmental events described in this review should encourage the perception of the eye not as a peripheral sensory organ that later connects with the brain, but as a continuous extension of the subcortical brain. The developing ventral diencephalon projects to the surface of the head to capture light stimuli and attracts back the RGC axons to receive the processed visual information.

## Author contributions

RP and TB framed the organization of the review and gathered literature. RP wrote the manuscript draft, generated tables and figures. RP and TB edited manuscript. All authors contributed to the article and approved the submitted version.
