# Supplementary material for: Signaling – transcription interactions in mouse retinal ganglion cells early axon pathfinding –a literature review
Source: Front Ophthalmol (Lausanne). 2023 May 17;3:1180142. doi: 10.3389/fopht.2023.1180142 (PMC11182120; doi:10.3389/fopht.2023.1180142)
Supplement: Supplementary file 1 [file Table_1.docx]

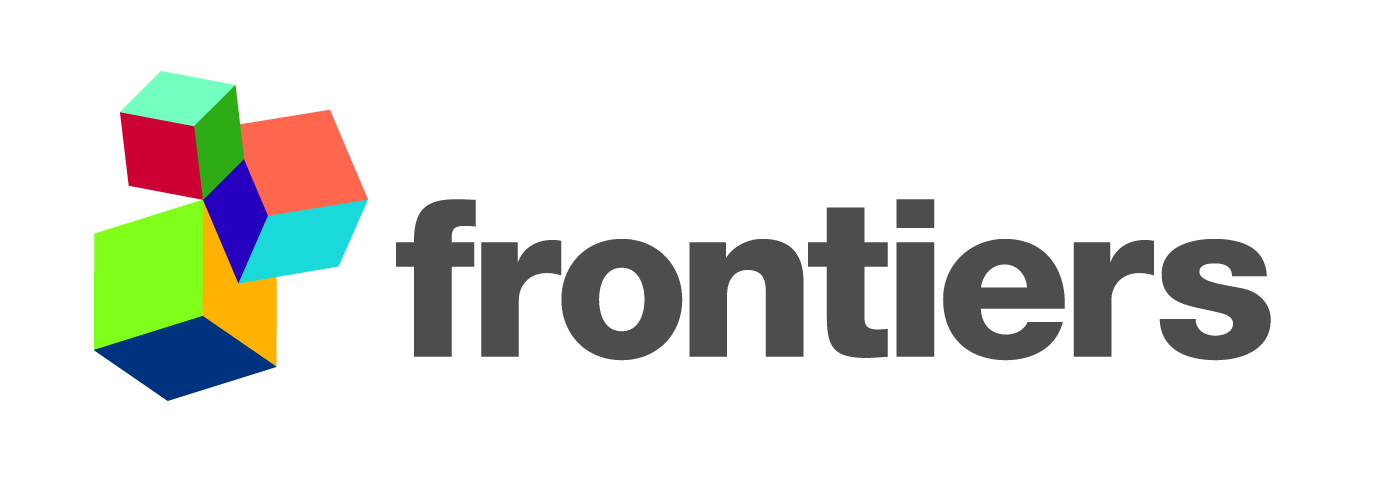


Supplementary Table 1

| (Giudice young PLUS mild1) AND Sajgo E15 RGC | Brooks E12 AND Sajgo E15 RGC | (Giudice young PLUS mild1) AND Sajgo E15 RGC AND Brooks E12 |
| --- | --- | --- |
| 'Ablim1' | 'Adcyap1' | Adcyap1' |
| 'Acot7' | 'Akap6' | 'Akap6' |
| 'Adcyap1' | 'Cntn2' | 'Cntn2' |
| 'Add2' | 'Dcc' | 'Dcc' |
| 'Afap1' | 'Dcx' | 'Dcx' |
| 'Akap6' | 'Ebf3' | 'Ebf3' |
| 'Apbb1' | 'Elavl3' | 'Elavl4' |
| 'Aplp1' | 'Elavl4' | 'Eomes' |
| 'Arl8a' | 'Eomes' | 'Igfbpl1' |
| 'Cacnb3' | 'Gng4' | 'Isl1' |
| 'Calm1' | 'Igfbpl1' | 'Myt1' |
| 'Ccl27a' | 'Irx2' | 'Nefl' |
| 'Chl1' | 'Isl1' | 'Nefm' |
| 'Cmip' | 'Mmp24' | 'Nfasc' |
| 'Cntn2' | 'Myt1' | 'Nrn1' |
| 'Coro1a' | 'Nefl' | 'Pou4f2' |
| 'Crip2' | 'Nefm' | 'Pou6f2' |
| 'Crmp1' | 'Nfasc' | 'Rgs4' |
| 'Ctsz' | 'Nhlh1' | 'Rph3a' |
| 'Dcc' | 'Nrn1' | 'Smim18' |
| 'Dclk1' | 'Pou4f2' | 'Sncg' |
| 'Dcx' | 'Pou6f2' | 'Stmn3' |
| 'Dner' | 'Rgs4' | 'Syt13' |
| 'Dpysl3' | 'Rph3a' | 'Thsd7b' |
| 'Dpysl5' | 'Scrt1' |  |
| 'Ebf1' | 'Smim18' |  |
| 'Ebf3' | 'Sncg' |  |
| 'Edil3' | 'Stmn3' |  |
| 'Elavl4' | 'Syt13' |  |
| 'Eomes' | 'Thsd7b' |  |
| 'Fxyd7' | 'Trhde' |  |
| 'Gap43' | 'Trim67' |  |
| 'Gnao1' | 'Tubb3' |  |
| 'Gng2' |  |  |
| 'Gng3' |  |  |
| 'Hspa12a' |  |  |
| 'Igf1' |  |  |
| 'Igfbpl1' |  |  |
| 'Isl1' |  |  |
| 'Islr2' |  |  |
| 'Kif1a' |  |  |
| 'Kif5a' |  |  |
| 'Kitl' |  |  |
| 'Klc1' |  |  |
| 'Klf7' |  |  |
| 'Klhl13' |  |  |
| 'Lingo1' |  |  |
| 'Ly6h' |  |  |
| 'Mapt' |  |  |
| 'Mgst3' |  |  |
| 'Mllt11' |  |  |
| 'Myt1' |  |  |
| 'Nefl' |  |  |
| 'Nefm' |  |  |
| 'Nfasc' |  |  |
| 'Ngfr' |  |  |
| 'Nrn1' |  |  |
| 'Nrp1' |  |  |
| 'Nsg2' |  |  |
| 'P2rx3' |  |  |
| 'Parvb' |  |  |
| 'Pcbp3' |  |  |
| 'Peli2' |  |  |
| 'Pfkp' |  |  |
| 'Pou4f2' |  |  |
| 'Pou6f2' |  |  |
| 'Rab6b' |  |  |
| 'Rbpms' |  |  |
| 'Reep1' |  |  |
| 'Reep5' |  |  |
| 'Reln' |  |  |
| 'Ret' |  |  |
| 'Rgs10' |  |  |
| 'Rgs4' |  |  |
| 'Rph3a' |  |  |
| 'Rpl13a' |  |  |
| 'Rtn1' |  |  |
| 'S100a10' |  |  |
| 'Sez6l2' |  |  |
| 'Slc18a2' |  |  |
| 'Smim18' |  |  |
| 'Snap91' |  |  |
| 'Snca' |  |  |
| 'Sncg' |  |  |
| 'Snhg11' |  |  |
| 'Stmn3' |  |  |
| 'Sv2a' |  |  |
| 'Synm' |  |  |
| 'Synpr' |  |  |
| 'Syt13' |  |  |
| 'Syt4' |  |  |
| 'Thsd7b' |  |  |
| 'Tmem130' |  |  |
| 'Tub' |  |  |
| 'Tuba1a' |  |  |
| 'Tubb2a' |  |  |
| 'Tubb2b' |  |  |
| 'Tubb4a' |  |  |
| 'Uchl1' |  |  |

**Supplementary Table 1.** Each column in the table is the result of logical intersections between gene lists identified in the specified samples/clusters by the studies included. The following logical operations have been performed: Giudice young PLUS mild1 AND Sajgo E15 RGC = Genes expressed in young RGCs at E15 = 99 genes; Sajgo E15 RGC AND Brooks E12 = Genes expressed in RGCs at E15 that are also expressed in E12 retinas = 32 genes; Giudice young PLUS mild1 AND Sajgo E15 RGC AND Brooks E12 = Genes expressed in young RGCs at E15 that are also expressed in E12 retinas = 24 genes.
