# Supplementary material for: Signaling – transcription interactions in mouse retinal ganglion cells early axon pathfinding –a literature review
Source: Front Ophthalmol (Lausanne). 2023 May 17;3:1180142. doi: 10.3389/fopht.2023.1180142 (PMC11182120; doi:10.3389/fopht.2023.1180142)
Supplement: Supplementary file 2 [file Table_2.docx]

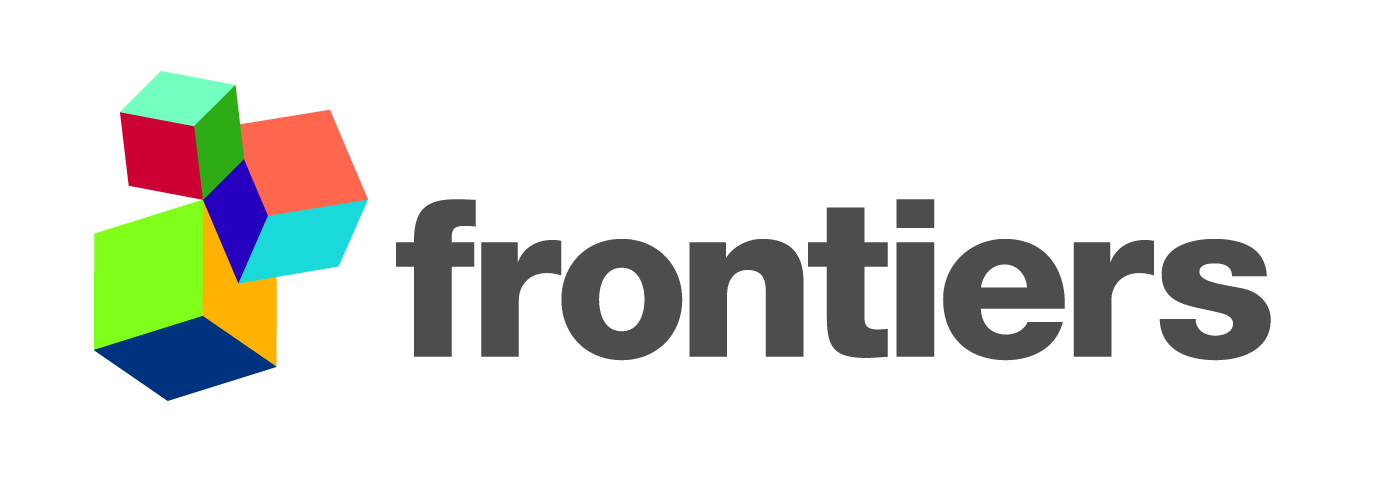


Supplementary Table 2

| Gene name | Extracellular space | Membrane | Cytoskeleton | Cytosol | Nucleus | Axon |
| --- | --- | --- | --- | --- | --- | --- |
| **App** |  |  |  | x |  | x |
| **Cdc42** |  | x | x |  |  |  |
| **Celsr3** |  | x |  |  |  |  |
| **Chl1** |  | x |  |  |  |  |
| **Cntn2** |  | x |  |  |  | x |
| **Dcc** |  | x |  |  |  | x |
| **Elavl4** |  |  |  | x |  | x |
| **Evl** |  |  | x |  |  | x |
| **Gap43** |  | x |  | x |  | x |
| **Igf1** | x | x | x |  |  |  |
| **Igfbpl1** | x |  |  |  |  |  |
| **Islr2** |  | x |  |  |  |  |
| **Kif1b** |  | x | x |  |  | x |
| **Kit** | x | x |  |  |  |  |
| **Kitl** | x | x | x |  |  | x |
| **Mmp24** |  | x |  |  |  | x |
| **Nrcam** |  | x |  |  |  | x |
| **Nrn1** |  | x |  |  |  |  |
| **Nrp1** |  | x |  |  |  | x |
| **Stmn2** |  | x |  | x |  | x |
| **Syt13** |  | x |  |  |  | x |
| **Tenm3** |  | x |  |  |  |  |
| **Trim67** |  |  | x |  |  | x |
| **Tubb3** |  |  | x |  |  | x |

**Supplementary Table 2.** Intracellular localization of the proteins encoded by each gene in Table 3. The “x” symbol indicates that the protein was found to be expressed in the compartment corresponding to the column according to <https://www.ncbi.nlm.nih.gov/gene/>.
